# Net, excess and absolute adsorption and adsorption of helium

**DOI:** 10.1007/s10450-016-9766-0

**Published:** 2016-02-01

**Authors:** Stefano Brandani, Enzo Mangano, Lev Sarkisov

**Affiliations:** Scottish Carbon Capture and Storage, School of Engineering, The University Edinburgh, The King’s Buildings, Mayfield Road, Edinburgh, EH9 3FB UK

**Keywords:** Adsorption equilibria, Net adsorption, Excess adsorption, Absolute adsorption, Helium adsorption

## Abstract

The definitions of absolute, excess and net adsorption in microporous materials are used to identify the correct limits at zero and infinite pressure. Absolute adsorption is shown to be the fundamental thermodynamic property and methods to determine the solid density that includes the micropore volume are discussed. A simple means to define when it is necessary to distinguish between the three definitions at low pressure is presented. To highlight the practical implications of the analysis the case of adsorption of helium is considered in detail and a combination of experiments and molecular simulations is used to clarify how to interpret adsorption measurements for weakly adsorbed components.

## Introduction

Despite several decades of research, there is still some confusion about what definition of adsorption (absolute, excess, net) to use for microporous materials and how to convert consistently between these properties. These issues have been recently brought in a particular spotlight due to the current ongoing interest in high pressure adsorption of weakly adsorbing gases, such as hydrogen, where the ambiguity in the definition of properties and procedures of conversion between them can lead to appreciable differences. As representative examples see: the comparison of adsorption of carbon dioxide on coal above 100 bar studied by different European laboratories (Gensterblum et al. 2010); the adsorption of carbon dioxide on semi-crystalline polymers (Lorenz and Wessling 2013); the adsorption of light gases on HKUST-1 (Moellmer et al. 2011) where two approaches for determining the absolute amount adsorbed are suggested. The discussion in the existing literature on this topic is extensive and an attempt to summarise it is not within the scope of this contribution. What, however, emerges from this discussion is that there is still no consensus on the form and standards according to which adsorption data should be reported; there is a still a significant confusion on what is actually directly measured in experiments; and there is lack of understanding of what information is needed for adsorption process modelling, leading to the diminished utility of many reported datasets. Most of the attention in the literature is devoted to different ways in which excess adsorbed amounts are defined and how to correct for helium adsorption, but the fact that these quantities do not allow the formulation of mass balances of adsorption processes seems largely missed.

In this article we aim to justify the reasoning behind the following points:

1. Do not use excess adsorption. This is not a well-defined property for microporous materials.
2. The way to quantify helium adsorption should be through adsorbed amounts on a volume basis vs density of the gas, leading to dimensionless Henry law constants, K.
3. Using dimensionless K values gives an immediate indication if it is necessary to distinguish between absolute, net or excess adsorbed amounts at low pressures.
4. Net adsorption is a useful, non-ambiguous means to report adsorption data.
5. To model adsorption processes absolute adsorption is needed and for microporous materials this requires the volume of the solid that includes the micropores.
6. The solid volume needed can be measured independently, thus allowing to convert net into absolute adsorbed amounts.

We limit our analysis only to materials which do not include mesopores and do not undergo structural changes. These additional cases can be addressed only once there is agreement on how to define adsorption in rigid microporous materials.

## Definitions of net, excess and absolute adsorption

To develop the correct macroscopic model of adsorption in a consistent thermodynamic framework it is always necessary to define clearly the system. What follows may appear slightly pedantic at times, but given the importance of finding a common basis to define adsorption equilibria we proceed with a step-by-step definition to avoid any misunderstanding.

**Fig. 1:**
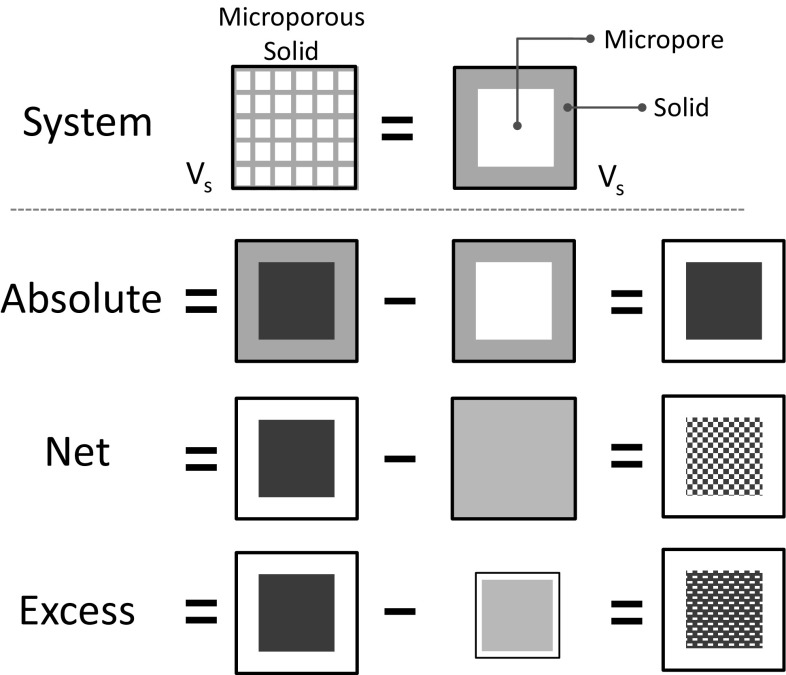
Definition of reference system and conceptual depiction of different adsorbed amounts

The system is defined as a rigid microporous solid, shown schematically in Fig. 1, as assumed by Myers and Monson (2014). This is the obvious definition of a system for absolute adsorption and it becomes effectively the system also in net and excess adsorption even when a volume external to the solid is considered since the effect of the external volume cancels out in the “net” and “excess” frameworks. For microporous solids the accumulation within the pores is very high compared to adsorption on the external surface, which is effectively negligible.

The system is in contact with an infinite reservoir of bulk fluid, which remains at constant temperature and pressure.

A fixed volume, *V*_*S*_, is defined which comprises the porous solid and the micropore volume. The total number of moles in the system is

1 $$n_{Tot} = n_{A} + n_{S}$$

where the suffix A indicates an adsorbate and S is the solid.

In absolute adsorption the solid is removed and

2 $$n^{abs} = n_{Tot} - n_{S} = n_{A}$$

In net adsorption the moles that would be in a fluid at the same pressure and temperature of the system with a concentration at equilibrium with the adsorbed phase that would occupy the volume of the system are removed.

3 $$n^{net} = n^{abs} - V_{S} c = n_{A} - V_{S} c$$

The total concentration can be written terms of the compressibility factor, *z*, which is equal to one for an ideal gas.

4 $$c = \frac{P}{zRT}$$

For the definition of the excess amount adsorbed one has to define the non-accessible volume, *V*_*NA*_

5 $$n_{{}}^{ex} = n_{{}}^{abs} - \left( {V_{S} - V_{NA} } \right)c = n_{A}^{{}} - \left( {V_{S} - V_{NA} } \right)c$$

For a microporous solid there are several ways in which the non-accessible volume can be defined:

1. The geometric volume of the solid;
2. The volume not accessible to the smallest adsorbate (or the adsorbate for a pure component);
3. The volume not accessible to a fixed probe molecule, typically chosen as helium.

In most cases the third option is the one commonly adopted and we will discuss some implications in a subsequent section. Intuitively, we anticipate that excess adsorption in micropores and disordered structures should be a more difficult property to define, compared to excess adsorption at a planar surface. However, as has been eloquently shown by Neimark and Ravikovitch (1997), even for a simple slit pore geometry, the concept of a geometric volume is associated with a number of ambiguities.

Given that for a porous solid *V*_*S*_ > *V*_*NA*_ it is possible to state that

6 $$n_{Tot}^{abs} > n_{Tot}^{ex} > n_{Tot}^{net}$$

The adsorbed phase concentration can be obtained by dividing the number of moles by the volume

7 $$q^{abs} = \frac{{n^{abs} }}{{V_{S} }} = \frac{{n_{A} }}{{V_{S} }} = q_{A}$$

and the equivalent net and excess concentrations are given by

8 $$q^{net} = \frac{{n^{abs} }}{{V_{S} }} - c = q_{A} - c$$

9 $$q^{ex} = \frac{{n^{abs} }}{{V_{S} }} - \frac{{V_{S} - V_{NA} }}{{V_{S} }}c = q_{A} - \varepsilon_{m} c$$

where the porosity of the microporous material is defined as

10 $$\varepsilon_{m} = \frac{{V_{S} - V_{NA} }}{{V_{S} }}$$

What is often not clear is that in the design of adsorption processes the basis of component mass balances is the concentration per unit volume, see for example Ruthven (1984) and chapter 16 in Perry’s manual (Le Van et al. 1997).

**Fig. 2:**
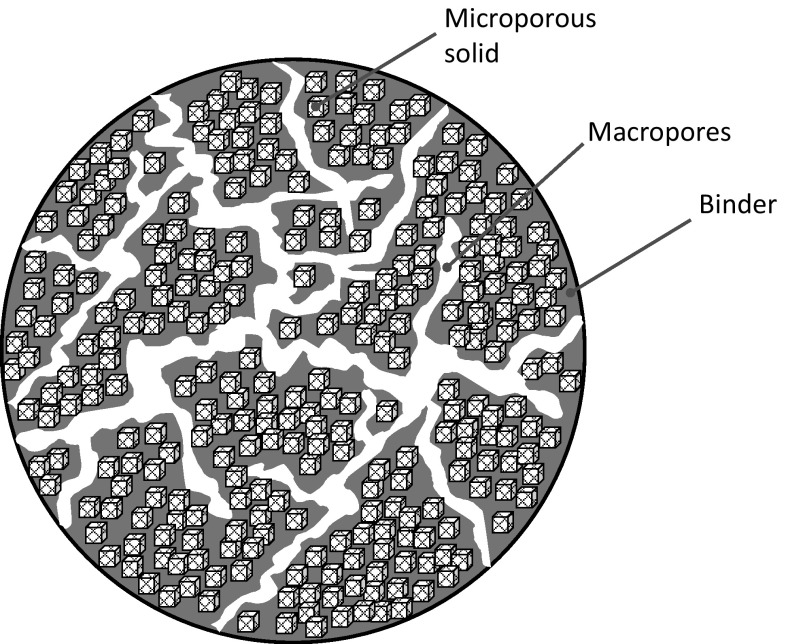
Particle including macropores, microporous solids and an inert binder

As an example consider a single pellet of volume *V*_*P*_, schematically shown in Fig. 2, in an uptake cell of volume *V*_*u*_. To determine the total number of moles in the system at equilibrium one has to know the volume of the macropores in the pellet which can be calculated from the macroporosity, *ε*_*P*_, and the fraction of active material in the pellet, *ϕ*_*C*_. The total number of moles in this system will be given by

11 $$n_{Tot} = \left( {V_{u} - V_{P} } \right)c + \varepsilon_{P} V_{P} c + \left( {1 - \varepsilon_{P} } \right)V_{P} \phi_{C} q^{abs}$$

The terms on the RHS are the moles in the gas phase; the moles in the macropores; and the moles adsorbed in the microporous material in terms of the adsorption isotherm which does not include the inert material. This can be rearranged into

12 $$n_{Tot} = \left( {V_{u} - V_{S} } \right)c + V_{S} \phi_{C} q^{abs}$$

This shows clearly that to formulate the mass balance, the volume of the particles which includes the micropores is needed.

If the adsorption isotherm is expressed in terms of the solid mass, *m* ^*abs*^, then the density of the solid which includes the micropores has to be included

13 $$n_{Tot} = \left( {V_{u} - V_{S} } \right)c + V_{S} \phi_{C} \rho_{S}^{C} m^{abs}$$

While one can develop a thermodynamic framework using *m* ^*abs*^ (see for example Myers and Monson 2014) it must be clear that in order to apply this framework to actual separations or gas storage systems one has to determine the density of the solid that includes the micropores. Clearly the two are interchangeable if this solid density is known.

From Eqs. 8 and 9 one can also express the absolute adsorbed amount in terms of excess and net adsorption and obtain an expression for the total adsorbed amount. What should be clear though is that to do this requires the knowledge of all the information needed to convert excess or net adsorption into absolute adsorption.

A further point that is useful to consider is that currently most researchers do not specify how the sample mass is measured. A notable exception is for example Hampson and Rees (1993) who describe in detail the use of saturated salt solutions to determine the wet sample mass of NaY zeolite and subsequent correction of the sample mass based on TGA measurements of water loss. A discussion of this point is included in Appendix 3.

## Correct limits at zero and infinite pressure

From the definitions given above it is possible to understand what the correct limiting behaviour for the different variables is at near-zero pressure. In this limit the absolute amount adsorbed can be described by Henry’s law and

14 $$q^{abs} = Kc$$

15 $$q^{net} = \left( {K - 1} \right)c = K^{net} c$$

16 $$q^{ex} = \left( {K - \varepsilon_{m} } \right)c = K^{ex} c$$

where the porosity of microporous materials will be 0 < ε_*m*_ < 1.

For most gas systems at relatively low temperatures the dimensionless Henry law constant, K, is typically » 1 and as a result it is often assumed as a reasonable approximation that in this limit all three definitions correspond to the same variable, i.e. *K* ≈ *K* ^*ex*^ ≈ *K* ^*net*^ within the uncertainty in experimental measurements. The use of the dimensionless Henry law constant is very useful in determining the actual importance of neglecting the difference between the three definitions at low pressures. It is in general not true that at low pressures all definitions yield the same result. It is more accurate to state that for any system the maximum absolute error obtained neglecting the difference in the definitions will be at most 1 for the dimensionless Henry law constant.

Clearly, for weakly adsorbed species the relative error may not be negligible and the maximum deviations will be observed for molecules such as helium or hydrogen. Therefore understanding helium adsorption in the Henry law region can provide useful insights into the importance of using absolute, excess or net adsorption.

For gas adsorption the energy of adsorption is negative, i.e. adsorption in microporous materials is exothermic. This implies that at high temperature K will tend to zero and that there will be a temperature at which the excess and the net adsorption are negative even at low pressures, i.e. *K*(*T*_*net*_) = 1 and *K*(*T*_*ex*_) = ε_*m*_, with *T*_*net*_ < *T*_*ex*_. We will revisit these relations from the statistical thermodynamics perspective in the “Molecular simulation” section.

What is less obvious is what happens close to infinite pressure, i.e. close to saturation. Consider the case of a single adsorbed component and for simplicity assume a spherical rigid molecule. For a bulk fluid the limiting density is equal to the close packing density which gives a dense phase fraction of (see for example Hales 2006)

17 $$\eta_{CP} = \frac{\pi }{{\sqrt {18} }} \approx 0.74048$$

This indicates that in a bulk fluid approximately 26 % of the volume is not occupied by the molecules.

Now by simple geometric considerations it is possible to argue that in a micropore it is unlikely that the molecules will be able to pack as densely due to the constraints imposed by the micropore walls. To understand this statement, consider the simple case of a long cylindrical pore with the same diameter as the spherical molecules. It is straightforward to calculate that in this configuration $\eta_{CP}^{A} = \frac{2}{3}$ since the molecules will be arranged as a string of pearls. Under confinement in a slit pore geometry a number of packing geometries is possible, however as has been systematically shown by Schmidt and Lowen (1997) and by Oguz et al. (2012), these packings have lower dense phase volume fraction than *η*_*CP*_. Thus, in general at infinite pressure the dense phase fraction of the adsorbed phase will be

18 $$\eta_{CP}^{A} < \eta_{CP}$$

It would be possible to define a reference volume to impose that the excess adsorbed amount at infinite pressure is zero (see for example Herrera et al. 2012) but this volume would be specific to each molecule and as a result issues of consistency would arise in multicomponent adsorption.

From the discussion above it is possible to derive the following properties at infinite pressure

19 $$q_{\infty }^{abs} = \frac{{\eta_{CP}^{A} }}{{\eta_{CP} }}\frac{{V_{S} - V_{NA} }}{{V_{S} }}c^{\infty } = q_{Sat}^{abs}$$

i.e. the saturation capacity of the micropores and

20 $$q_{\infty }^{net} = \left( {\frac{{\eta_{CP}^{A} }}{{\eta_{CP} }}\varepsilon_{m} - 1} \right)c_{{}}^{\infty } = q_{Sat}^{abs} - c_{{}}^{\infty } < 0$$

21 $$q_{\infty }^{ex} = \left( {\frac{{\eta_{CP}^{A} }}{{\eta_{CP} }} - 1} \right)\varepsilon_{m} c_{{}}^{\infty } = q_{Sat}^{abs} - \varepsilon_{m} c_{{}}^{\infty } < 0$$

Therefore qualitatively the absolute adsorbed amount will increase monotonically to the saturation capacity, while the net and excess adsorbed amounts will initially increase and then go through a maximum.

For excess and net adsorption the correct limit at infinite pressure is always negative. This indicates that for each property, excess and net adsorption, there is a pressure point at which the property is zero. For the excess adsorbed amount this is termed the Bering point (Neimark and Ravikovitch 1997) and the equivalent point for net adsorption will be at a lower pressure.

**Fig. 3:**
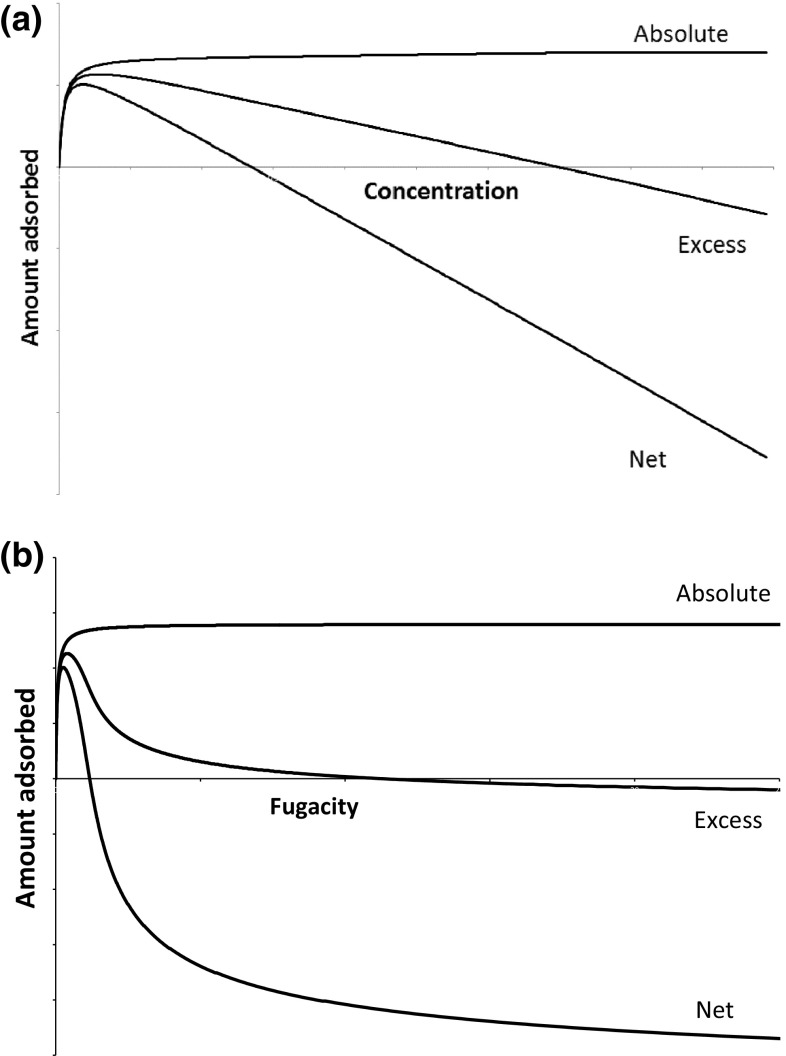
Qualitative behaviour of absolute, excess and net adsorption **a** versus concentration or density; **b** versus fugacity or pressure

This analysis of the limiting behaviours shows that for microporous materials while there is always only one value of the absolute adsorbed amount corresponding to a pressure or fugacity, both the net and excess adsorbed amounts may have two corresponding pressure or fugacity values and are not strictly positive. The qualitative behaviour of absolute, excess and net adsorbed amounts is shown in Fig. 3 which is obtained using a Langmuir isotherm coupled with a Reidlich–Kwong cubic equation of state. The cubic equation of state has the correct limit at infinite pressure for the compressibility factor (Brandani and Brandani 2007) which results from a finite density in this limit.

From Fig. 3 it is possible to observe that the shape of absolute adsorption vs concentration or fugacity remains the same. This is not the case for excess and net adsorbed amounts and this is due to the fact that for an equation of state that includes a finite density at infinite pressure, both excess and net adsorption will show an inflection at higher fugacities, which is not present in the plot vs concentration (or density).

These observations imply that the natural variable to choose for a thermodynamic treatment of adsorption is the absolute adsorbed amount, a point strongly advocated in the recent review by Myers and Monson (2014). The fact that absolute adsorption is the obvious thermodynamic variable but cannot be measured directly should not come as a surprise. Often in thermodynamics fundamental properties are not directly measurable, think for example of fugacity and chemical potential which can be derived from clear definitions of reference states and form the basis upon which we commonly define fluid phase equilibria (Prausnitz et al. 1999).

## What can be measured and what should be reported

In principle at least, it is possible to measure directly the absolute adsorbed amount on a solid mass basis using for example impedance spectroscopy (Keller and Staudt 2005) or NMR techniques (see for example Banas et al. 2005). In molecular simulation of adsorption, it is always the absolute amount adsorbed that is calculated and all common analytical adsorption isotherms are formulated for absolute adsorption. These are the adsorption isotherms used in adsorption process simulators.

As we discuss below in the most common experimental adsorption techniques absolute adsorbed amounts cannot be measured directly. It is also often stated in the literature that it is the excess amount adsorbed that is measured (directly) in experiments (see for example Sircar 1999; Myers and Monson 2002). This is, however, somewhat misleading as both net and excess adsorbed amounts require an additional experiment, typically a helium expansion measurement, to derive the actual values. In the case of net adsorbed amounts only one extra measurement is needed if the cell geometry does not change.

What is still needed is the volume of the solid *V*_*S*_ that includes the micropores or the corresponding solid density, in order to be able to use the results in models of adsorption units.

Since the majority of adsorption measurements are carried out with 3 techniques—volumetric; chromatographic; and gravimetric—it is useful to discuss these in greater detail.

**Fig. 4:**
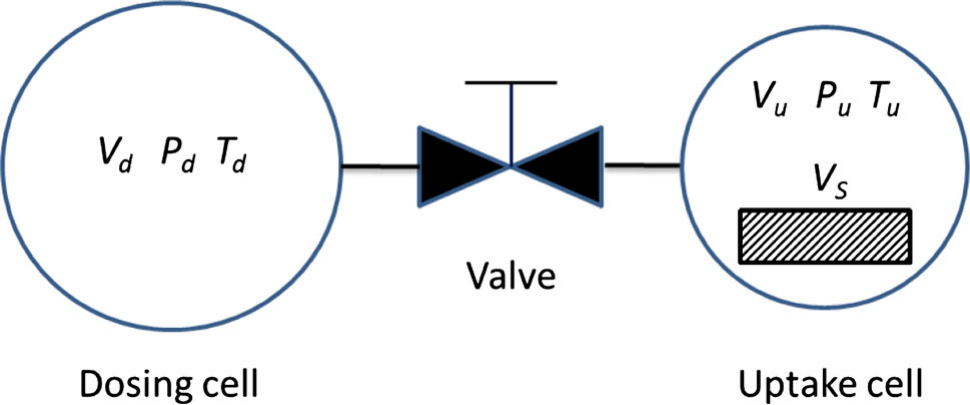
Schematic diagram of a volumetric system

A schematic diagram of a volumetric system is shown in Fig. 4. In this experiment a known amount of gas is added to a calibrated dosing cell. The valve is opened so that the dosing cell is now connected to the uptake cell, which contains the adsorbent, and the final equilibrium pressure is measured.

The measurement of the pressures before and after opening the valves is combined with the knowledge of the volumes of the dosing and uptake cells and a mass balance is applied using the temperatures of the two cells to determine the gas densities. The total number of moles in the system before and after the valve is opened is given by

22 $$n_{Tot} = \left( {V_{u} - V_{S} } \right)\frac{{P_{u}^{0} }}{{z_{u} RT_{u} }} + V_{d} \frac{{P_{d}^{0} }}{{z_{d} RT_{d} }} + V_{S} q_{0}^{abs}$$

23 $$n_{Tot} = \left( {V_{u} - V_{S} } \right)\frac{{P_{u}^{1} }}{{z_{u} RT_{u} }} + V_{d} \frac{{P_{d}^{1} }}{{z_{d} RT_{d} }} + V_{S} q_{1}^{abs}$$

with $P_{u}^{1} = P_{d}^{1}$ once equilibrium is achieved.

Clearly none of the three definitions of adsorbed amounts are actually measured. If the volume of the solid is not known a priori, the same system is used to carry out a helium expansion experiment and the volume measured is then used to estimate the excess adsorbed amount. It is possible to measure net adsorption if experiments without the solid are carried out to calibrate the total volume of the system and

24 $$n_{Tot} = V_{u} \frac{{P_{u}^{1} }}{{z_{u} RT_{u} }} + V_{d} \frac{{P_{d}^{1} }}{{z_{d} RT_{d} }} + V_{S} q_{1}^{net}$$

One can argue that measuring net adsorption in this system is less ambiguous than estimating an excess adsorbed amount. It is useful to note that this is the quantity of interest in gas storage, since what one is trying to maximise is the total number of moles in a system with an adsorbent at a given temperature and pressure compared to the system without the adsorbent. If gas storage is not the only application of interest, or if one needs to develop a kinetic model of the system even for gas storage, then one needs the value of the specific volume of the solid to be able to use the experimental data to obtain the absolute adsorbed amount.

**Fig. 5:**
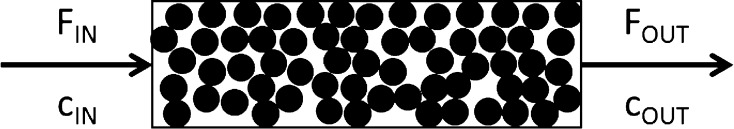
Schematic diagram of a chromatographic experiment

Figure 5 shows a schematic diagram of a chromatographic or breakthrough experiment. Here a gas flows into the system and at time zero either a pulse of adsorbate is added to the carrier gas (chromatographic experiment) or the system is perturbed by a step change in concentration (breakthrough experiment). What should be measured are both the outlet concentration and the volumetric flowrates (Mason and Buffham 1996), which then allow one to determine through a mass balance the difference in the amount of gas that enters and exits the system:

25 $$V_{F} \frac{d}{dt}\left( {\frac{\mathop \smallint \nolimits cdz}{L}} \right) + V_{S} \frac{d}{dt}\left( {\frac{\mathop \smallint \nolimits qdz}{L}} \right) = \left( {Fc} \right)_{IN} - \left( {Fc} \right)_{OUT}$$

The terms on the LHS are the accumulation in the fluid and solid phases respectively, where the integrals yield the average gas and solid phase concentration along the column length, *L*. In practice often the volumetric flowrate at the outlet is calculated from the concentration and the inlet carrier flowrate (Malek and Farooq 1997) and the flowrate corrections may become very important for large step changes in concentration, especially for desorption experiments (Brandani 2005; Wang et al. 2011). The general assumption for single adsorbates (Ruthven 1984) is that the carrier is inert and not adsorbed, which for most systems at low pressure is valid if helium is used as the carrier gas. Brandani (2005) includes the correction for adsorption of the carrier gas using a Henry law constant to account for this contribution. In general a full dynamic simulation can be used to interpret the experimental results, which would require the simultaneous solution of at least two mass balances (see for example Friedrich et al. 2015).

In chromatographic or breakthrough experiments it is also true that none of the three adsorbed amounts is measured directly. One can perform helium expansion experiments (see for example Talu et al. 1996) and estimate the excess adsorbed amount in a similar way as for the volumetric system. In this experiment one could also use a large molecule which is size-excluded from the micropores (for example trimethylbenzene or mesitylene in the case of silicalite) at high temperature to determine the specific volume of the solid and calculate absolute adsorbed amounts. If empty column experiments are performed it is in principle possible to determine the net adsorbed amount, although to be accurate systems with very low pressure drops should be used (either large beads or very low flowrates) since in the mass balance the accumulation in the void space of the column will vary with pressure.

**Fig. 6:**
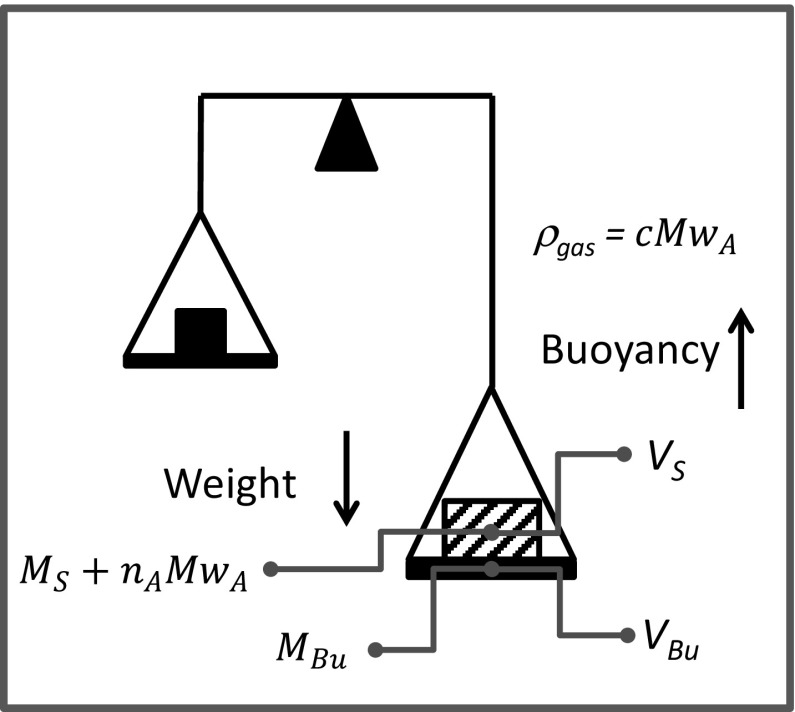
Schematic diagram of a gravimetric system

In a gravimetric system what is measured is the force acting on the sample in a configuration schematically show in Fig. 6. In this case the adsorbed amounts are determined by a force balance and not a mass balance. The measured force is the resultant of the weight of the bucket (or sample holder), the weight of solid and adsorbate minus the buoyancy which is acting on the volume of the solid that includes the micropores and the volume occupied by the bucket. Implicit in this is the assumption that if the balance is in a flow system either the drag force is negligible or more accurately that the difference of the drag force with and without the sample is negligible, if experiments without the sample are performed to calculate the correction for the buoyancy and drag force due to the bucket.

The resultant force measured by the balance (without drag from a moving fluid) is given by

26 $${{\varOmega }} = \left( {M_{Bu} + M_{S} + n_{A} Mw_{A} } \right)g - \left( {\varepsilon_{m} V_{S} + V_{NA} + V_{Bu} } \right)cMw_{A} g$$

If *ɛ*_*m*_ *V*_*S*_ + *V*_*NA*_ = *V*_*S*_ is known, then the experimentally determined resultant force can be converted into an absolute adsorbed amount. If only the volume and mass of the bucket are used to correct the reading from the balance then net adsorption is calculated.

27 $$\frac{{{{\varOmega }} - \left( {M_{Bu} + M_{S} } \right)g + V_{Bu} cMw_{A} g}}{{M_{S} }} = \frac{{n_{A} Mw_{A} }}{{M_{S} }}g - \frac{{V_{S} }}{{M_{S} }}cMw_{A} g = \frac{{V_{S} }}{{M_{S} }}q^{net}$$

Helium experiments can be carried out on the same system to estimate the non-accessible volume and the corresponding excess adsorbed amount.

As a summary, it is clear that in general net adsorption can be measured and that its definition is not ambiguous for microporous solids. Nevertheless absolute adsorption is the variable needed in order to develop appropriate equilibrium and kinetic models of adsorption units. To convert net adsorption to absolute adsorption all that is needed is the density of the solid on the basis of the total volume of the solid. Excess adsorbed amounts are not measured directly and require the same effort if not more as that needed to measure net adsorption.

*V*_*S*_ is the volume which includes the micropores and cannot be measured directly in the experimental setups used to determine adsorption equilibria, but the solid density defined on the basis of this volume is needed to use the equilibrium data in kinetic models and process simulations. Therefore, it is useful to give an indication of how this quantity should be measured.

The most direct measurement (see for example Pini 2014) is achieved using a mercury porosimeter (Lowell et al. 2006). Given that at the mechanical equilibrium micropores are too small to allow mercury to enter and a mercury intrusion experiment is carried out over a time which will not allow further equilibration, the volume will be measured with excellent accuracy. Alternatively for microporous materials synthesised with a template one could perform a measurement with a helium or water pycnometer before and after the template is removed (in fact what is needed is only the measurement with the template and the mass of the sample after the template is removed to calculate the correct solid density). Another alternative to estimate V_S_ is to carry out cryogenic adsorption experiments, preferably with argon, to determine the micropore volume, combining this with the pycnometry results. For crystalline structures, such as zeolites and microporous MOFs, V_S_ can be obtained directly from the crystallographic data. What should be clear is that the emphasis should be on defining how to measure or calculate V_S_ accurately and not continue with further efforts to define the non-accessible volume for the determination of the excess adsorbed amount, which is not the thermodynamic or practical variable of interest.

## Helium adsorption

From the points considered so far, it is clear that weakly adsorbed components are the ones for which most problems will arise. A practically important example is that of helium adsorption, since in addition to being a system of interest in some applications it is used routinely to determine the skeletal density of microporous materials after synthesis. Adsorption of helium in microporous solids at close to room temperature is very weak and therefore allows to understand clearly the differences between absolute, excess and net adsorption. Excess amounts adsorbed at high pressure are routinely reported and quantifying helium adsorption would also allow us to assess uncertainties associated with these data. It may also shed some light on the reported discrepancies between different research groups, particularly in the cases of weakly adsorbing molecules.

If we consider a microporous solid, an assumption is often made that helium does not adsorb at temperatures around 300 K, so that a volume expansion experiment with helium can be used to determine the skeletal density of the material. This assumption does not imply that no helium molecules enter the micropores. A statement that in fact would be more accurate is that the density of helium in the micropores above room temperature does not differ from the density of helium in the bulk gas phase, i.e. that for helium at these relatively high temperatures the excess amount adsorbed is zero. If this is true then the helium experiment will measure the actual skeletal density of the solid and in order for this to be the case clearly $n_{He}^{A} \ne 0$ and at low pressures

28 $$q_{He}^{abs} = \frac{{V_{S} - V_{NA} }}{{V_{S} }}c_{He} = \varepsilon_{m} c_{He} = \frac{{\varepsilon_{m} }}{zRT}P$$

or simply that the dimensionless Henry law constant of helium, when its excess adsorbed amount is zero, is in fact the microporosity of the sample and will be in the range 0.1–0.6 for most systems, even at high temperatures.

To demonstrate that excess helium adsorption cannot be zero except at a single temperature we start with the more accurate assumption that at sufficiently high temperatures and relatively low pressures the absolute adsorption of helium can be described using Henry’s law.

As shown in Appendix 1 it is possible to derive the following relationship for the temperature dependence of the dimensionless Henry law constant

29 $$\left. {\frac{dlnK}{dT}} \right|_{{q_{A} }} = \left. {\frac{dlnK}{dT}} \right|_{{{\psi }}} = - \frac{{s_{G} - s_{A} }}{RT} = - \frac{{{{\Delta }}U}}{{RT^{2} }}$$

If excess adsorption is assumed to be zero, then from Eq. 16

30 $$K = \varepsilon_{m}$$

and the dimensionless Henry law constant is independent of temperature, therefore one must have

31 $$s_{G} = s_{A}$$

By simple physical reasoning, the molar entropy in the gas phase cannot be the same as that of the molecules confined inside the micropores and intuitively the following must be true:

32 $$s_{G} > s_{A}$$

and gas adsorption in micropores is exothermic. Thus, the condition given by Eq. 31 cannot be obeyed on these simple fundamental grounds, which in turn implies dependence of the dimensionless Henry law constant on temperature, according to Eq. 29. Equation 29 shows that at very high temperatures the absolute amount adsorbed will tend to zero; both excess and net amounts adsorbed will be negative at low pressures. This shows even further that the use of helium adsorption to define excess adsorbed amounts leads to ambiguity, because the apparent skeletal density will depend on the temperature at which the helium experiment is carried out.

The temperature dependence of helium adsorption is an issue that is not new (Maggs et al. 1960; Springer et al. 1969). To correct for this in the determination of the skeletal density of microporous materials the initial approach was to assume zero adsorption at a high temperature and then determine iteratively the Henry law constant of helium as a function of temperature assuming a constant isosteric heat of adsorption (Suzuki et al. 1987). Sircar (2001) modified the approach of Suzuki et al. (1987) suggesting that it would be more accurate to use low temperature data to determine the temperature dependence of the Henry law constant. Gumma and Talu (2003) proposed an improvement over Sircar’s method by removing the assumption that helium adsorption was zero at any temperature and through an iterative procedure determined the correction volume and applied it to their gravimetric data of helium adsorption on HISIV 3000. All these approaches are based on the assumption that the isosteric heat of adsorption at zero loading is independent of temperature and this is not strictly true.

## Molecular simulations

In molecular simulations of adsorption, it is always the absolute amount adsorbed that is calculated. The issue then becomes to convert the simulated absolute adsorption values to the excess values in a procedure consistent with the experiments:

33 $$q_{sim}^{ex} = \frac{{n_{sim}^{abs} }}{{V_{S} }} - \frac{{V_{S} - V_{NA, sim} }}{{V_{S} }}c = q_{sim}^{A} - \varepsilon_{m,sim} c$$

One can also determine the net adsorbed amount from the molecular simulations which is given by

34 $$q_{sim}^{net} = \frac{{n_{sim}^{abs} }}{{V_{S} }} - c = q_{sim}^{A} - c$$

We note that there is no ambiguity in the definition of the simulated net adsorbed amount as pointed out by Gumma and Talu (2010).

Earlier theoretical studies sought the definition of the non-accessible volume *V*_*NA,sim*_ based on the geometric definition of the pore and solid structure. However, as has been discussed by Neimark and Ravikovitch (1997), this leads to a number of ambiguities even when a simple pore model, such as a slit pore, is considered. To avoid these ambiguities the authors argued that porosity should be measured in a way analogous to the experimental procedure, or in other words using computational helium porosimetry. The dimensionless Henry’s constant can be easily computed according to:

35 $$K_{sim} = RTK_{P} = \frac{{\int_{{V_{S} }} {\exp ( - {{U(r)} \mathord{\left/ {\vphantom {{U(r)} {kT}}} \right. \kern-0pt} {kT}})dV_{S} } }}{{V_{S} }} = \left\langle {e^{ - U(r)/kT} } \right\rangle$$

where *U*(*r*) is the interaction energy of the helium atom with the porous material at position *r*, *k* and *R* are the Boltzmann and gas constants respectively, and the last property in brackets is the average Boltzmann factor which can be easily obtained using the Widom insertion method. Similarly to the experiments, the dimensionless Henry law constant of helium adsorption provides the microporosity of the sample, when its excess adsorbed amount is zero. In fact, this approach based on computed helium volume has now become a standard procedure in comparison of simulated and experimental isotherms (Talu and Myers 2001), while the calculation itself has been implemented in several porous structure characterization packages (Sarkisov and Harrison 2011).

From the statistical-mechanical point of view, expressions (15) and (16) can be related to the solid–gas second virial coefficient, providing a link between adsorption and solid–fluid interactions. Indeed, the second virial coefficient is given by:

36 $$B_{2} = \int_{{V_{S} }} {\left( {\exp ( - {{U(r)} \mathord{\left/ {\vphantom {{U(r)} {kT}}} \right. \kern-0pt} {kT}}) - 1} \right)dV_{S} }$$

It is easy to see, therefore, that

37 $$B_{2} = \int_{{V_{S} }} {\left( {\exp ( - {{U(r)} \mathord{\left/ {\vphantom {{U(r)} {kT}}} \right. \kern-0pt} {kT}}) - 1} \right)dV_{S} } = \int_{{V_{S} }} {\exp ( - {{U(r)} \mathord{\left/ {\vphantom {{U(r)} {kT}}} \right. \kern-0pt} {kT}})dV_{S} } - V_{S} = V_{S} (K_{sim} - 1)$$

And consequently:

38 $$q_{sim}^{net} = \frac{{B_{2} }}{{V_{S} }}c$$

In statistical thermodynamics, the temperature at which *B*_2_ = 0 is called Boyle’s temperature. In the application to adsorption problems, it will be the temperature at which net adsorption is zero. For excess adsorption, a similar expression can be obtained:

39 $$q_{sim}^{ex} = \left( {K_{sim} - \varepsilon_{m} } \right)c = \left( {\frac{{B_{2} }}{{V_{S} }} + 1 - \varepsilon_{m} } \right)c$$

Again, it is easy to see that there should a single value of temperature at which the expression in brackets on the right is equal to zero. Finally we note the argument here largely follows that of Neimark and Ravikovitch (1997) in their work, however, the zero value of the second virial coefficient (Boyle’s temperature) corresponded to zero excess adsorption. This is because within their definition of the system based on slit pore geometry, *ε*_*m*_ = 1.

## Case study: helium adsorption in silicalite

To probe the statements and the analysis above we consider, as a case study, adsorption of helium in silicalite. The details of the calculations involved in Eq. 35, including parameters of the force field, are provided in Appendix 2.

**Fig. 7:**
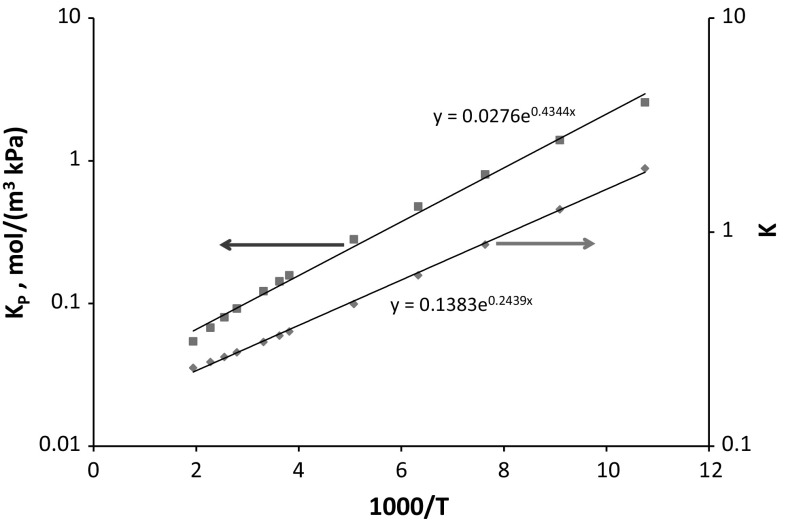
van’t Hoff plot of predicted Henry law constants of He in silicalite. *Trend lines* obtained from all points show average Δ*U* = −2.0 kJ/mol and Δ*H* = −3.6 kJ/mol

Dimensionless Henry’s constants for helium in silicalite are calculated at the same values of temperature considered by Gumma and Talu (2003). If we treat the simulation results in the same way in which experiments are used, the next step would be to correlate the data with a constant heat of adsorption. Figure 7 shows the van’t Hoff plot of the predicted values for both *K*_*P*_ and *K*. One important result that can be observed from this plot is that the adsorption energy, Δ*U*, is to a very good approximation nearly constant over a wide range of temperatures. If for example we consider only 5 points at the lowest and highest temperatures the adsorption energy is −2.1 and −1.7 kJ/mol respectively. These values should be compared with −2.0 kJ/mol obtained from all the data points. From molecular simulations, the adsorption energy can be obtained explicitly and it decreases from −2.2 kJ/mol at 93 K to −1.5 kJ/mol at 515 K.

If the adsorption energy is effectively constant then the isosteric heat, Δ*H*, will not be constant. This is because helium is weakly adsorbed and the adsorption energy is of the same order of magnitude as *RT* in the temperature range 150–300 K. For helium in NaA zeolites an isosteric heat of 4 kJ/mol was reported by Vashchenko and Katalnikova (1996). From the values of the dimensionless Henry law constant it is also clear that in the case of helium (*K* in the range 0.2–2) absolute, net and excess adsorbed amounts will be significantly different at low pressures. Using dimensionless *K* values, Boyle’s temperature ($q_{sim}^{ex}$ = 0) is estimated at 120 K. The fractional porosity ε_*m*_ = 0.307 is calculated according to Eq. 35 at 300 K, and therefore this is, trivially, the temperature at which $q_{sim}^{ex}$ = 0 according to Eq. 39. As expected (see discussion above) *T*_*net*_ < *T*_*ex*_. Alternatively, the fractional porosity could be estimated using some other means. For example, First et al. (2011) reported a silicalite fractional porosity of 0.45 based on purely geometric considerations (point probe) and 0.405 based on a rigid sphere with diameter of 2 Å. The corresponding temperatures where $q_{sim}^{ex}$ = 0 will be therefore 202 and 225 K, respectively. However, we emphasize again that the calculations based on this porosity will be inconsistent with the experiments, as this property suffers from essentially the same ambiguities as discussed by Neimark and Ravikovitch (1997) in the context of a slit pore geometry.

**Fig. 8:**
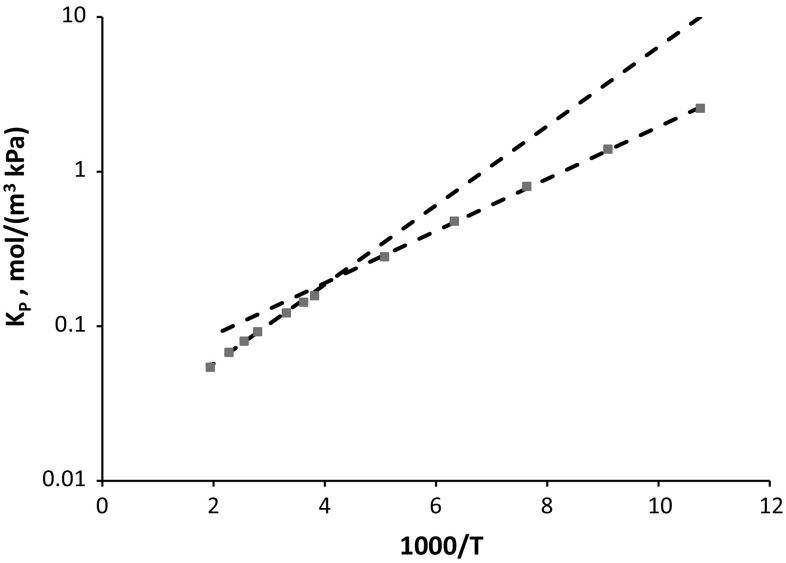
van’t Hoff plot of the predicted Henry law constant showing the temperature dependence estimated using five points at the lowest (Δ*H* = −3.2 kJ/mol) and the highest (Δ*H* = −4.9 kJ/mol) temperatures. From molecular simulations, the Δ*H* increases from −3.0 kJ/mol at 93 K to −5.76 kJ/mol at 515 K.

Figure 8 shows that the approach of Suzuki et al. (1987) and the improvements of Sircar (1999) and of Gumma and Talu (2003), which are based on the assumption that Δ*H* is independent of temperature, are not fully accurate and confirms that this assumption is not valid especially at higher temperatures (see also Do et al. 2008).

Given that the adsorption energy inevitably has a smaller temperature dependence as seen from the analysis above, an improved method to determine the correction for the adsorption of helium would be based on plotting the data in a van’t Hoff plot of dimensionless Henry law constants assuming to a good approximation that the adsorption energy is independent of temperature.

## Comparison with the data of Gumma and Talu (2003)

As a final demonstration that combining standard measurements it is possible to overcome any ambiguity in the interpretation of adsorption equilibrium experiments we predict helium adsorption in HISIV 3000 for which good quality data are available (Gumma and Talu 2003). The data were reported including all the information needed to calculate the adsorbed amounts from the actual signal measured from the microbalance.

Firstly, we recognise that HISIV 3000 is a commercial silicalite available in pelletized form from UOP, a Honeywell company. The material has a binder and therefore the results from the molecular simulations cannot be used directly without an estimate of the fraction of binder in the material.

In our laboratory we have used mercury porosimetry to obtain the density of the particles which includes the micropores, and combined helium pycnometry and the results from the molecular simulations to estimate the fraction of binder. The experimental details are reported in Appendix 3.

The apparent skeletal density decreases slightly with increasing temperature in agreement with the fact that the helium adsorbed decreases leading to an apparent increase in the non-accessible volume.

Considering the binder a non-porous inert solid, the fraction of the crystals in the pellets can be calculated from

36 $$\phi_{C} = \left( {\frac{1}{{\rho_{S} }} - \frac{1}{{\rho_{Sk} }}} \right)\frac{{\rho_{S} }}{{\varepsilon_{m} }}$$

Using the average values from Tables 1 and 2 it is possible to estimate *ϕ*_*C*_ ≅ 0.77, i.e. that the binder is approximately 23 % of the solid volume.

**Table 1:** Summary of the results of the mercury porosimetry analysis on HISIV 3000

Mass (g)	Pellet density (g/cc)	V_macro_ (cc/g)	V_solid_ (cc/g)	Solid density (g/cc)
1.011	1.140	0.337	0.540	1.85
1.002	1.155	0.329	0.537	1.86

**Table 2:** Summary of the results of the helium pycnometry analysis on HISIV 3000

Temperature (°C)	Mass (g)	V_ave_ (cc/g)	σ_vol_	Skeletal density (g/cc)
14.2	3.187	1.297	0.0008	2.457
19.5	3.187	1.314	0.0013	2.425
30.3	3.184	1.324	0.0038	2.406

The raw data, (Ω − *M*_*S*_ − *M*_*Bu*_)/*M*_*S*_, reported by Gumma and Talu (2003) can be converted into net adsorbed amounts using

37 $$q^{net} = \left( {\frac{{{{\varOmega }} - M_{S} - M_{Bu} }}{{M_{S} }}\frac{1}{{Mw_{He} }} - \frac{{V_{Bu} }}{{M_{S} }}c} \right)\rho_{S}$$

knowing that 5.2574 g of sample was used for data at 197 K and below and 5.5744 g was used at the higher temperatures (Gumma 2015, personal communication). At the lower pressures

38 $$q^{net} = \left( {K\phi_{C} - 1} \right)c$$

**Fig. 9:**
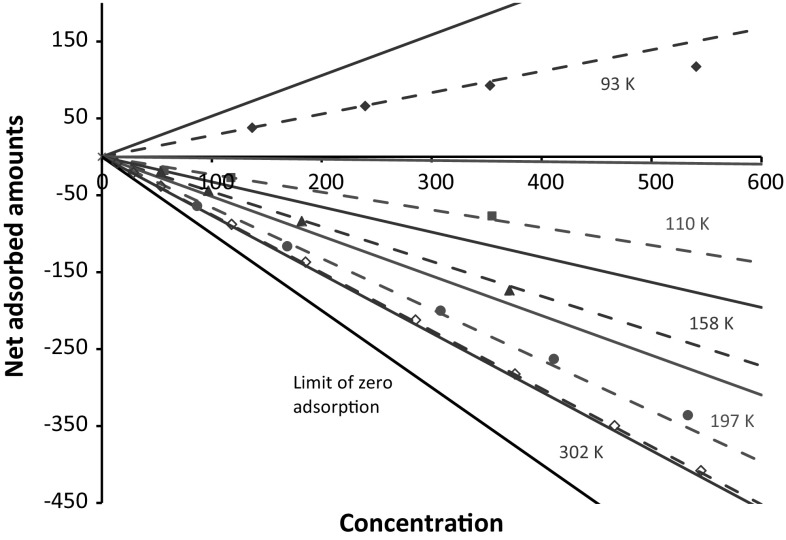
Comparison of net adsorbed amounts at 93, 110, 197 and 302 K. Predictions shown as continuous lines and symbols are data from Gumma and Talu (2003). Dashed lines are calculated from Henry law constants determined from the data

Figure 9 shows the comparison of the original data presented as net adsorption and the corresponding molecular simulations at relatively low pressures where the data are close to the Henry law region. On this plot the data at the lowest temperature appear to be furthest apart, but one should consider that the experimental dimensionless Henry law constant is 1.66 while the predicted one is 1.99 and the difference is magnified by plotting net adsorbed amounts.

**Fig. 10:**
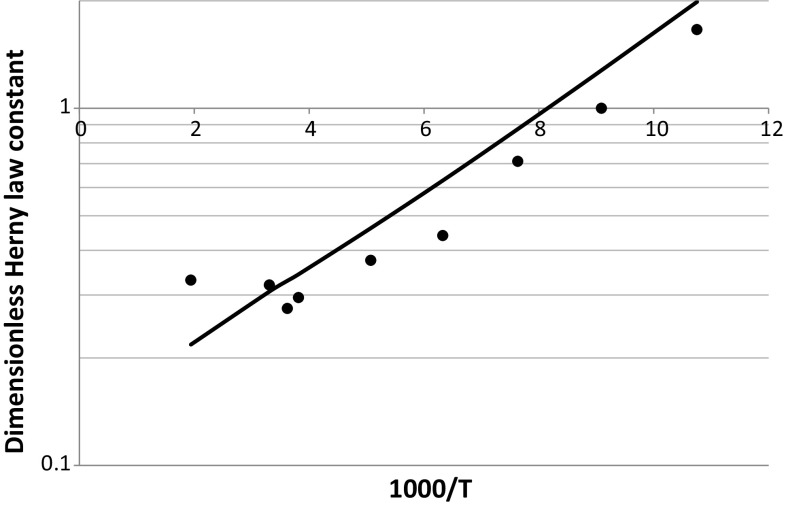
van’t Hoff plot of dimensionless Henry law constants derived from the data of Gumma and Talu (2003) (*symbols*) and calculated from molecular simulations (*continuous line*)

The fact that the predictions are in acceptable agreement can be seen clearly from Fig. 10 which shows the comparison of the dimensionless Henry law constants. The molecular simulations reproduce to a high degree of accuracy the adsorption energy. One could adjust the force field parameters to improve the match to the data, but there is some uncertainty in the comparison to be expected given that our sample of HISIV 3000 may differ from the one used by Gumma and Talu (2003). Alternative parameters for helium and silicalite have been proposed by Tomar et al. (2011) which have been used to describe the results of Gumma and Talu (2003), but these authors did not recognise the fact that HISIV 3000 pellets are not a pure crystalline material.

The experimental results show some system effect at the higher temperatures, where the experimental apparatus is probably reaching its limit of detection. The data above 302 K should be treated with caution.

## Conclusions

Based on the results obtained it is possible to arrive at some final reflections and recommendations.

Net and excess adsorbed amounts are not directly measured but in most cases can be obtained with an additional experiment using the same instrument. This is the main reason why it is common to report either excess or net adsorption but for the development of thermodynamic and kinetic models one needs the absolute adsorbed amount, therefore it would be more useful if the solid density (including the micropores) was measured and absolute adsorbed amounts were reported directly. If this is not possible, because the laboratory is not equipped to carry out the additional measurement, then it would be less ambiguous to report net adsorption compared to excess adsorption. It is the opinion of the authors that excess adsorbed amounts should not be used, but in recognising that there may still be a preference to report excess adsorbed amounts by some groups, it is important to emphasize that the non-accessible volume used should be clearly reported with the data, i.e. that the data are reported in a way that net adsorption can be calculated easily.

We have shown that in general representing adsorbed amounts vs concentration or density of the gas phase gives curves which are more easily interpreted. Representing data in this way, allows at low pressures the determination of the dimensionless Henry law constant. This variable provides a very simple check to confirm if it is necessary to distinguish between the three definitions of adsorbed amounts since the maximum deviation between the three definitions is one. As a simple example, consider the adsorption of nitrogen and oxygen in 5A zeolite data of Ruthven and Xu (1993). The dimensionless Henry law constant (in beads) for oxygen at 303 K is 14.6, which shows that for the determination of the absolute adsorbed amount the maximum error due to the volume correction would be less than 7 % but not negligible. The authors report also a value of 10,500 for nitrogen at 174 K. Clearly this shows that for example when using nitrogen adsorption at around liquid nitrogen temperatures to characterise porous adsorbents the distinction between the three definitions of adsorption is negligible and well within experimental uncertainty.

Molecular simulations of helium adsorption on silicalite have shown that the adsorption energy can be considered as independent of temperature to a good degree of approximation. This indicates that in order to determine accurately the skeletal density of a material one should use the dimensionless Henry law constant to correct for the helium adsorbed at the temperature of the experiment since the isosteric heat will vary with temperature more substantially.

We have also shown that combining helium pycnometry and mercury porosimetry it is possible to define both the density of pelletized materials and estimate the fraction of binder, thus allowing direct comparison of gravimetric data and molecular simulations. This also confirmed that the correct density of a microporous material can be determined from mercury porosimetry and allows us to calculate absolute adsorbed amounts from net adsorbed amounts.

The renewed interest in energy efficient adsorption separation processes and gas storage applications, including cases of light gases at high pressures, coincides with co-current unprecedented developments in the material chemistry, where thousands of new porous materials are discovered and reported every year. This also led to a wealth of adsorption data being published and to take a full advantage of these data, there is a clear need for some standardization. The important matter to consider is that models of adsorption processes require absolute adsorbed amounts and the density of the microporous solid which includes the volume of the micropores. We conclude that more emphasis should be given to finding different reliable ways in which the correct density of microporous solids can be measured in addition to mercury porosimetry.

## Appendix 1

Starting from the basic equations that define the Gibbs energy for a system composed of one adsorbate (A) and a solid (S)

39 $${\text{G}} = n_{A} \mu_{A} + V_{S} \mu_{S}$$

where for the solid a solid volume basis is assumed which can be interchanged with a mass basis through the appropriate solid density. At equilibrium the fluid phase chemical potential is

40 $$\mu = \mu_{A}$$

The differential of the Gibbs energy is given by

41 $${\text{dG}} = - SdT + VdP + \mu_{A} dn_{A} + \mu_{S} dV_{S}$$

From the total differential of the Gibbs energy, Eq. 39, the Gibbs–Duhem equation is obtained

42 $$0 = - SdT + VdP - n_{A} d\mu_{A} - V_{S} d\mu_{S}$$

To define absolute adsorption the state of the solid without adsorbate has to be defined

43 $${\text{G}}^{0} = V_{S} \mu_{S}^{0}$$

and

44 $${\text{dG}}^{0} = - S^{0} dT + V^{0} dP + \mu_{S}^{0} dV_{S}$$

and the corresponding Gibbs–Duhem equation

45 $$0 = - S^{0} dT + V^{0} dP - V_{S} d\mu_{S}^{0}$$

By combining the two Gibbs–Duhem equations we have

46 $$V_{S} d\left( {\mu_{S} - \mu_{S}^{0} } \right) = - \left( {S - S^{0} } \right)dT + \left( {V - V^{0} } \right)dP - n_{A} d\mu_{A}$$

The additional assumption that in our system the quantity of solid does not change is introduced and hence the system is at constant volume, i.e. V = V^0^ = V_S_. Therefore

47 $$d\left( {\mu_{S} - \mu_{S}^{0} } \right) = - \frac{{\left( {S - S^{0} } \right)}}{{V_{S} }}dT - q_{A} d\mu_{A}$$

where $q_{A}^{{}} = \frac{{n_{A} }}{{V_{s} }}.$ Under isothermal conditions this results into the Gibbs isotherm:

48 $$d{{\psi }} = - d\left( {\mu_{S} - \mu_{S}^{0} } \right) = q_{A}^{{}} d\mu_{A} = q_{A}^{{}} RTdlnf$$

where ψ is the grand potential.

For the gas phase

49 $$d\mu = - s_{G} dT + v_{G} dP$$

While for the adsorbed phase rearranging Eq. 47 and using the definition in Eq. 48

50 $$d\mu_{A} = \frac{1}{{q_{A} }}d{{\psi }} - \frac{{\left( {S - S^{0} } \right)}}{{q_{A} V_{S} }}dT$$

But along the equilibrium curve the equivalent of the Clausius–Clapeyron equation is obtained

51 $$\frac{1}{{q_{A} }}d{{\psi }} - \frac{{\left( {S - S^{0} } \right)}}{{q_{A} V_{S} }}dT = - s_{G} dT + v_{G} dP$$

Which shows that at constant grand potential

52 $$\left. {\frac{dP}{dT}} \right|_{{{\psi }}} = \frac{{s_{G} }}{{v_{G} }} - \frac{{\left( {S - S^{0} } \right)}}{{v_{G} q_{A} V_{S} }}$$

and defining

53 $$s_{A} = \frac{{\left( {S - S^{0} } \right)}}{{q_{A} V_{S} }}$$

it is possible to obtain in the low pressure region (*z* = 1)

54 $$\left. {\frac{dlnP}{dT}} \right|_{{{\psi }}} = \frac{{s_{G} - s_{A} }}{RT}$$

If Henry’s law is valid

55 $$q_{A} = Kc = \frac{K}{{v_{G} }} = K_{P} P$$

and from the Gibbs adsorption isotherm

56 $$d{{\psi }} = q_{A} v_{G} dP = KdP$$

or

57 $${{\psi }} = KP = K_{P} RTP = RTq_{A}$$

which gives

58 $$0 = K\left. {\frac{dP}{dT}} \right|_{{{\psi }}} + P\left. {\frac{dK}{dT}} \right|_{{{\psi }}} {\text{or the equivalent }}0 = \left. {\frac{dlnP}{dT}} \right|_{{{\psi }}} + \left. {\frac{dlnK}{dT}} \right|_{{{\psi }}}$$

and combining this with Eq. 54 one has

59 $$\left. {\frac{dlnK}{dT}} \right|_{{{\psi }}} = - \frac{{s_{G} - s_{A} }}{RT}$$

Alternatively the isosteric heat, i.e. the heat obtained if the adsorbed amount is constant, can be derived. In this case *dq*_*A*_ = 0 which combined with Eq. 55 yields

60 $$0 = K_{P} \left. {\frac{dP}{dT}} \right|_{{q_{A}^{{}} }} + P\left. {\frac{{dK_{P} }}{dT}} \right|_{{q_{A}^{{}} }} {\text{or the equivalent }}0 = \left. {\frac{dlnP}{dT}} \right|_{{q_{A}^{{}} }} + \left. {\frac{{dlnK_{P} }}{dT}} \right|_{{q_{A}^{{}} }}$$

From Eq. 57

61 $$\left. {\frac{d\phi }{dT}} \right|_{{q_{A} }} = Rq_{A}$$

and combining Eqs. 51, 53 and 61 one obtains

62 $$\left. {\frac{dlnP}{dT}} \right|_{{q_{A} }} = \frac{{s_{G} - s_{A} + R}}{RT}$$

or

63 $$\left. {\frac{{dlnK_{P} }}{dT}} \right|_{{q_{A} }} = - \frac{{s_{G} - s_{A} + R}}{RT}$$

By simple algebraic manipulations one can also show that

64 $$\left. {\frac{{dlnK_{P} }}{dT}} \right|_{{{\psi }}} = - \frac{{s_{G} - s_{A} + R}}{RT}$$

and

65 $$\left. {\frac{dlnK}{dT}} \right|_{{q_{A} }} = - \frac{{s_{G} - s_{A} }}{RT}$$

Defining the isosteric heat as

66 $$\Delta H = \left( {s_{G} - s_{A} } \right)T + RT$$

and the adsorption energy as

67 $$\Delta U = \left( {s_{G} - s_{A} } \right)T = \Delta H - RT$$

Then

68 $$\left. {\left. {\frac{{dlnK_{P} }}{dT}} \right|_{{q_{A} }} = \frac{{dlnK_{P} }}{dT}} \right|_{{{\psi }}} = - \frac{\Delta H}{{RT^{2} }}$$

69 $$\left. {\left. {\frac{dlnK}{dT}} \right|_{{q_{A} }} = \frac{dlnK}{dT}} \right|_{{{\psi }}} = - \frac{\Delta U}{{RT^{2} }}$$

While the final relationships can be found in the literature, the very important point is that we have demonstrated that the two definitions of heats are *independent* of the fact that the molar volume of the adsorbed phase can be neglected when compared to that of the gas phase, i.e. the definition of the system on the basis of the volume that includes micropores and non-accessible solid effectively eliminates the molar volume of the adsorbed phase if the solid can be considered an inert.

## Appendix 2

The technical details of the calculations required to obtain dimensionless Henry’s constant for helium in silicalite are presented. The calculation was performed using an approach based on finely discretized (0.2 Å) lattice representation of the simulation cell, as described in Sarkisov (2012).

Interactions between helium atom and oxygen and silicon atoms of the structure are described using the Lennard-Jones potential. Lennard-Jones parameters for these atoms are summarized in Table 3.

**Table 3:** Lennard-Jones parameters

Atom	ε/k (K)	σ (Å)	Reference
He	10.9	2.640	Hirschfelder et al. (1954)
O	72.2	3.265	Talu and Myers (2001)
Si	0.0	0.0	Commonly adopted convention

Interactions parameters for atoms of different types are obtained using the standard Lorentz–Berthelot mixing rules. Interactions were cut-off at 13 Å, no long tail corrections were applied.

## Appendix 3

Prior to each experiment the HISIV 3000 sample was subjected to overnight regeneration at 250 °C under vacuum in an outgassing station of an Autosorb iQ. The sample mass and the mercury porosimeter cell masses were measured using a Mettler Toledo XS205 DualRange balance with an accuracy of 0.1 mg.

The details of the measurement of the sample mass are often not included in publications, but our experience with training several students and postdocs is that often this is the source of some uncertainty. One can simply take a sample and measure it on the balance, but with an adsorbent one has to consider that air will adsorb on the sample and if the material is hydrophilic the uncertainty in the sample mass may not be negligible. The standard approach in our laboratory is to use the outgassing station of an Autosorb iQ as described above and backfill with helium, place a cap on the cell and measure the weight of the cell. This may not be feasible if one is packing 1 kg in a breakthrough apparatus or 1 mg in a ZLC experiment and not all laboratories are equipped with balances in enclosures that allow to control the atmosphere. Therefore, it would be useful if the actual procedure to measure the sample mass was defined more clearly.

Table 4 gives the weights measured on HISIV 3000 for different procedures: (a) regeneration followed by backfill as above; (b) sample poured out and back into the cell (less than 1 min) and plug inserted; (c) sample allowed to equilibrate with ambient air.

**Table 4:** Weight of the HISIV 3000 sample and cell

	(a), g	(b), g	(c), g
Empty cell + plug	13.5485	13.5554	13.5554
Cell + sample + plug	16.7126	16.7315	16.8209
Sample	3.164	3.176	3.271

With a hydrophobic sample such as HISIV 3000 the uncertainty is less than 0.5 % if the sample is handled rapidly. The uncertainty increases to 3 % if the sample is left for longer times in air. Things are different with a hydrophilic material, such as 13X. In our laboratory the normal procedure for hydrophilic materials is to carry out a TGA experiment and quantify the amount of water adsorbed in a manner similar to Hampson and Rees (1993), but not with salt solutions as the adsorbed amount at room temperature is near saturation even at very low partial pressures of water. There is also a cross-check of results from different instruments to ensure consistency. To quantify the range of uncertainty Table 5 shows the sample masses on UOP 13X APG 8 × 12 commercial beads obtained from: (a) sample taken from sealed container with no regeneration and put rapidly in the cell with a plug; (b) sample regenerated at 275 °C in the outgassing station as above (i.e. with helium backfill); (c) sample poured rapidly (less than 30 s) out of the cell and back in the cell (i.e. with air) and plug inserted; (d) sample allowed to equilibrate with ambient air.

**Table 5:** Weight of the 13X sample and cell

	(a), g	(b), g	(c), g	(d), g
Empty cell + plug	13.5598	13.5471	13.5556	13.5598
Cell + sample + plug	16.8119	16.7037	16.7297	17.5603
Sample	3.252	3.157	3.174	4.001

If the sample is not regenerated the error is 3 %, but clearly given the significant increase at equilibrium, 27 %, the mass of a sample exposed to air will depend on how the measurement is done, i.e. the care taken by the person preparing the experiment.

The measurements of the density of the HISIV 3000 sample were carried out using an Autosorb Poremaster mercury porosimeter. The first step is the measurement of the weight of the empty sample cell, this is then loaded in the instrument to be completely filled with mercury, and the total weight is measured (sample cell + mercury). Finally, in a similar way, the sample is loaded in the cell and filled completely with mercury. From these weights the densities are determined. Once inside the instrument a traditional porosimetry analysis can be carried out and from the total volume of mercury intruded the macroporosity of the samples can be extracted. Prior to the experiment the sample was outgassed as described above. Two runs were carried on different samples and the results are summarised in Table 1.

The skeletal density was measured experimentally using a Quantachrome UltraPyc 1200e He pycnometer. The sample mass used was 3.18 g, this ensured that approximately half the volume of the sample cell was occupied by the sample. To maximise the accuracy of the measurements a careful calibration procedure of the instrument was used for each experimental temperature. NIST certified spheres were used to determine accurately the volume of 84 1/8 in. stainless steel beads, giving a total reference volume of 1.408 cm^3^. At the temperature of the experiment, measured directly by the instrument and controlled using a thermostated bath connected to the pycnometer, the cell volumes were recalibrated with the known volume of steel beads ensuring that the helium expansion volume would be very accurate given the small difference between the calibration and the measured volumes.

Once the HISIV 3000 material is loaded into the sample cell, a purge step of 2 h under vacuum was carried out using an oil free Edwards nXDS 6i vacuum pump. This ensured both the thermal equilibration of the sample as well as the full evacuation of the pores of the sample. After the purge the experiment proceeds by running a sequence of 10 volume measurements; the resulting final volume measured is calculated as the average of the last 5 runs. Table 2 shows the results of the series of experiments carried out at different temperatures.

## List of symbols

*B*_2_: Second virial coefficient (m^3^)
*c*: Gas phase concentration (mol m^−3^)
*c*^∞^: Fluid phase density at infinite pressure (mol m^−3^)
*f*: Fugacity (kPa)
*F*: Volumetric flowrate (m^3^ s^−1^)
*g*: Gravitational acceleration (m s^−2^)
*G*: Gibbs energy (J)
*G*^0^: Gibbs energy of the solid without the adsorbate (J)
$\Delta H$: Adsorption enthalpy (J mol^−1^)
*k*: Boltzmann constant (J atom^−1^ K^−1^)
*K*: Dimensionless (absolute) Henry law constant
*K*^*ex*^: Dimensionless excess Henry law constant
*K*^*net*^: Dimensionless net Henry law constant
*K*_*P*_: Henry law constant (absolute) (mol m^−3^ kPa)
*L*: Length of adsorption column (m)
*M*_*Bu*_: Mass of bucket (kg)
*M*_*S*_: Mass of solid (kg)
*Mw*_*A*_: Molecular weight of adsorbate (kg mol^−1^)
*n*_*A*_: Moles of adsorbate (mol)
*n*^*abs*^: Absolute adsorbed amount (mol)
*n*^*ex*^: Excess adsorbed amount (mol)
*n*^*net*^: Net adsorbed amount (mol)
*n*_*S*_: Moles of solid (mol)
*n*_*Tot*_: Total moles in the system (mol)
*P*: Pressure (kPa)
$P_{d}^{0}$: Pressure in dosing cell before valve is opened (kPa)
$P_{d}^{1}$: Pressure in dosing cell after valve is opened (kPa)
$P_{u}^{0}$: Pressure in uptake cell before valve is opened (kPa)
$P_{u}^{1}$: Pressure in uptake cell after valve is opened (kPa)
*q*_*A*_: Adsorbed phase concentration (mol m^−3^)
*q*^*abs*^: Absolute adsorbed phase concentration (mol m^−3^)
*q*^*ex*^: Excess adsorbed phase concentration (mol m^−3^)
*q*^*net*^: Net adsorbed phase concentration (mol m^−3^)
*r*: Position (m)
*R*: Ideal gas constant (J mol^−1^ K^−1^)
*s*_*A*_: Molar entropy of adsorbed phase (J mol^−1^ K^−1^)
*s*_*G*_: Molar entropy of gas phase (J mol^−1^ K^−1^)
*T*: Temperature (K)
*U*: Interaction energy of atom (J atom^−1^)
$\Delta U$: Adsorption energy (J mol^−1^)
*v*_*G*_: Molar volume of gas phase (m^3^ mol^−1^)
*V*_*Bu*_: Volume of bucket (m^3^)
*V*_*d*_: Volume of dosing cell (m^3^)
*V*_*F*_: Volume of fluid phase (m^3^)
*V*_*NA*_: Volume not accessible (m^3^)
*V*_*P*_: Volume of pellet (m^3^)
*V*_*S*_: Volume of solid, including micropores (m^3^)
*V*_*u*_: Volume of uptake cell (m^3^)
*z*: Compressibility factor

## Greek letters

*ɛ*_*m*_: Porosity of microporous material
*ɛ*_*P*_: Macroporosity of pellet
*ϕ*_*C*_: Fraction of active material in a pellet
*μ*_*A*_: Chemical potential of adsorbate (J mol^−1^)
*μ*_*S*_: Chemical potential of solid (volume basis) (J m^−3^)
$\mu_{S}^{0}$: Chemical potential of solid without adsorbate (J m^−3^)
*η*_*CP*_: Reduced density at close packing
$\eta_{CP}^{A}$: Reduced density of adsorbed phase at close packing
*ρ*_*Gas*_: Density of gas phase (kg m^−3^)
*ρ*_*S*_: Solid density including micropores (kg m^−3^)
$\rho_{S}^{C}$: Density of active material (kg m^−3^)
*ρ*_*Sk*_: Skeletal density (kg m^−3^)
ψ: Grand potential (J m^−3^)
Ω: Signal from microbalance (force) (N)
